# Menarche, Menstruation, Menopause and Mental Health (4M): a consortium facilitating interdisciplinary research at the intersection of menstrual and mental health

**DOI:** 10.3389/fgwh.2023.1258973

**Published:** 2023-08-29

**Authors:** Gemma C. Sharp, Luana De Giorgio

**Affiliations:** ^1^ School of Psychology, University of Exeter, Exeter, United Kingdom; ^2^ Public Health and Sports Science, Exeter Medical School, University of Exeter, Exeter, United Kingdom

**Keywords:** menstruation, mental health, interdisciplinary research, women’s health, menstrual health, biopsychosocial approach

## Abstract

Menstrual and mental health form a close relationship that is under-appreciated in scientific research, clinical practice and social policy. This association is extremely complex, involving interactions between biology, psychology and social, political and structural influences on health and wellbeing. Research in these areas has traditionally been siloed: focusing on menstrual or mental health in isolation, or the interrelation from a limited one-dimensional perspective. We recognised the need for a more holistic and comprehensive approach that considers the complex interweaving nature of menstrual and mental health. In 2021, we established the Menarche, Menstruation, Menopause and Mental Health (4M) consortium as a tool to address this gap and to facilitate interdisciplinary research. This paper provides a comprehensive source of information about 4M for researchers and stakeholders who may be interested in joining or working with the consortium.

## Introduction

Experiences and conditions that disproportionately affect women are under-researched (1), and although there is growing public interest and discussion about menstruation and menstrual health, research is lagging behind (2, 3). Menstrual experiences intersect with physical, mental, and social wellbeing, which are all recognised as important facets for good health (4, 5). For example, menstrual and mental health form a close relationship that is under-appreciated in scientific research, clinical practice and social policy. Menstrual disorders such as endometriosis and polycystic ovarian syndrome (PCOS), and symptoms such as irregular periods, heavy menstrual bleeding and severe pain, are associated with lower quality of life and wellbeing, non-attendance at school and work, and higher rates of mental health disorders (4, 6–11). Some people with mental health conditions such as depression, psychotic disorders, borderline personality disorder, or bipolar disorder, report that their mental health symptoms are exacerbated during certain cycle phases (e.g., premenstrual exacerbation) (12–14). An estimated 20%–40% of people who menstruate experience premenstrual syndrome (PMS), and a further 2%–8% have severe, disabling premenstrual symptoms that meet the clinical definition for premenstrual dysphoric disorder (PMDD) (15).

The association between menstrual and mental health is extremely complex, involving interactions between biology, psychology and social, political and structural influences on health and wellbeing. Adding to the complexity, this relationship and the underlying mechanisms change throughout the lifecourse and in different contexts. For example, around the time of the first menstrual period (menarche), physical, cognitive and social changes can impact body image and self-esteem (16, 17), and earlier age at menarche has been associated with a higher risk of depression in late adolescence (18). At the other end of the reproductive lifecourse, hormonal changes, physical symptoms and psychosocial factors during the menopausal transition can affect mood and depressive symptoms in some individuals (19, 20). Menstrual symptoms are often more problematic at these stages too, for example cycles can be more irregular and individuals may experience heavier bleeding (21). In addition, the relationship between menstrual and mental health may be modified by socioeconomic and cultural context. For example, poorer access to menstrual products and clean facilities can introduce or exacerbate the impact of (problematic) menstrual symptoms on mental health (5, 22, 23). Furthermore, social stigma and taboo around menstruation is present across many cultures and could exacerbate any adverse relationship with mental health (4, 24).

The intersection of menstrual and mental health is severely under-appreciated and under-researched, leaving millions of people with limited support and treatment options. Research in these areas has traditionally been siloed; focusing on menstrual or mental health in isolation, or the interrelation from a limited one-dimensional perspective. There has been limited collaboration between disciplines, leading to fragmented and incomplete knowledge about the interplay between these health domains. A comprehensive understanding of the complex intersection of menstrual and mental health, and how to reduce any adverse relationship, requires integration of multiple perspectives and methods.

## Menarche, Menstruation, Menopause and Mental Health (4M) consortium

We recognised the need for a more holistic and comprehensive approach that considers the complex interweaving nature of menstrual and mental health. In 2021, we established the Menarche, Menstruation, Menopause and Mental Health (4M) consortium to address this need by facilitating interdisciplinary research. The consortium brings together researchers from a range of disciplines, including psychology, medicine, sociology, policy studies, genetics, and epidemiology, to discuss and work together on interdisciplinary research projects and advance knowledge in this important area. By fostering collaboration and networking among researchers, the 4M consortium provides a platform for researchers to share ideas, knowledge, and methods, leading to a more comprehensive understanding.

## The 4M vision and mission

The overarching vision of 4M is a world in which menstrual experiences, including events around menarche and menopause, do not adversely affect mental health and social wellbeing. We believe that decisions and practices about individuals' menstrual and mental health should be informed by scientific understanding and listening to the needs and experiences of those individuals. Therefore, our mission is to facilitate interdisciplinary, stakeholder-informed, impact-focused, inclusive research at the intersection of menstrual and mental health. The research we facilitate aims to develop a better understanding of:

• The biological, psychological, social, and environmental mechanisms that link menstrual and mental health;
• How interventions can effectively target these mechanisms to improve the relationship between menstrual and mental health;
• How these mechanisms and interventions affect menstrual and mental health differently in different contexts and at different stages of the lifecourse, from menarche to menopause.

## The 4M approach

We facilitate research by introducing and connecting academic researchers from and across multiple disciplines. This includes early career researchers, who we try to support to develop and sustain their careers in this burgeoning interdisciplinary field. We also build collaborations with non-academic stakeholders (for example, patients, healthcare providers, charities, and policy makers), who can provide a unique insight into research need, facilitate co-production and Patient and Public Involvement and Engagement (PPIE) informed research, and aid pathways to impact. Table 1 provides an overview of how our activities map to our approach.

**Table 1:** The 4M approach: why and how.

The 4M approach	Why we take this approach	How we achieve this via our activities
Interdisciplinary academic collaboration	• The drivers of menstrual and mental health are multifaceted, complex, and interacting • This requires an interdisciplinary approach to draw on multiple perspectives and triangulate evidence from different methods and data source	• 4M is open to researchers from any discipline • Monthly online meetings and a newsletter to keep in touch • Occasional in-person meetings to help connect • Seminar series to share knowledge • Collaborative grant applications • Collaborative research papers
Collaboration with non-academics	• Non-academic stakeholders can provide a unique insight into the research need • This can help researchers to design, interpret and prioritise research that matters • Stakeholders can facilitate pathways to impact—disseminating research directly to patients and to relevant actors to bring about change	• Database of stakeholders • Stakeholder workshops • Co-produced research projects and grant applications • Public-facing information on our website, social media, and through our seminar series
Supporting early career researchers (ECRs)	• The field is under-appreciated and under-researched, so it provides fertile ground for ECRs to establish their careers • However, for the same reason, there are limited funding opportunities and fewer senior researchers, so ECRs may require more support • The field needs a long-term approach—supporting ECRs to sustain their interest and activity over time	• No seniority policy on membership • Opportunities for ECRs to lead workshops • Cross-4M supervision of PhD students • Financial support for ECRs to attend meetings • Workshops targeting ECRs to provide skills for careers in this field

## Funding, establishing, and growing 4M

4M was established using a Generator Award from the GW4 Alliance, a network of universities in the South West of the UK (University of Bath, University of Bristol, Cardiff University, University of Exeter), and was initially known as the GW4 Menstrual and Mental Health Research Community (GW4MMHRC). Dr Gemma Sharp devised the idea for the consortium and approached existing GW4 collaborators and identified new collaborators via the websites of each GW4 university. The initial funding application included 19 researchers and the funding period was from June to November 2021. The funding covered direct costs associated with our activities, including an in-person two-day event, admin support, facilitation of online workshops, live illustration of our online workshops, logo design and website hosting. At the end of the funding period, following consultation with all members, the name was changed from GW4MMHRC to 4M for simplicity and to reflect the focus of our research more accurately.

In December 2022, GW4 awarded 4M an additional Development Award to allow us to employ a part-time research assistant (Luana de Giorgio) to support our activities. Following this enhanced capacity, we opened membership of 4M to researchers outside of the GW4 universities. At the time of writing, 4M has over 130 academic members across more than 50 institutions and 13 countries. Our diversity strengthens our ability to facilitate and conduct interdisciplinary research.

## Identifying and developing 4M's research focus

4M launched with a series of online scoping workshops in which members shared their current research projects and expertise, followed by a facilitated discussion of how we could combine our interests to work together. From these workshops, we identified five themes and then held further online workshops to generate research questions and ideas relevant to each theme. All our workshops were live illustrated by illustrator Laura Sorvala (www.laurasorvala.com; examples on the 4M website www.4mhealth.uk). The illustrations serve two purposes: firstly, as a concise aide-memoire for 4M researchers; and secondly, as an accessible output that we share with other researchers and non-researchers via email, our website, and our Twitter page. We also developed an animation to summarise our research questions under each theme, and used these accessible outputs to invite stakeholders to feedback on our ideas through an online form and a series of hybrid stakeholder workshops. The themes and examples of initial questions are presented in Table 2.

**Table 2:** Initial research themes and questions.

Theme	Examples of initial research questions
Mental health and wellbeing throughout the menstrual cycle	• How do physiological and emotional symptoms fluctuate throughout the menstrual cycle? • How can we best measure this? • Are effects of (hormonal) menstrual phase on mood and emotions mediated by effects on physiology? • How do individuals with mental health disorders experience the menstrual cycle? When and why are mental health symptoms exacerbated? • What are the barriers to diagnosis of PMDD? What is the average time and journey to diagnosis? • How do findings vary by socioeconomic, demographic, global, and cultural context?
Adolescent experiences and education	• What is the mechanism through which early menarche influences risk of depression in adolescence? • How do individuals with a mental health disorder experience menarche? • Does the timing and quality of school-based sexual and reproductive health education affect mental health? • What is the public understanding of menstruation and the reproductive lifecourse and how can this be improved? • How do findings vary by socioeconomic, demographic, global, and cultural context?
Menopause and mental health	• What are the predictors of poor mental health around menopause? • What is the impact of menopause on cognition and dementia? • How do individuals with a mental health disorder experience menopause? • What are the best treatments to prevent or reduce negative effects of the menopause transition and/or (peri-)menopausal symptoms on mental health? • How do findings vary by socioeconomic, demographic, global, and cultural context?
Menstrual health disorders and problematic symptoms	• Are people who experience problematic menstrual symptoms at a higher risk of mental health disorders? (And are people with a mental health disorder at a higher risk of experiencing problematic menstrual symptoms?) • Do problematic menstrual symptoms causally affect mental health? In which individuals? Or are they correlated because of (confounding) genetic, physiological or environmental factors that influence both menstrual symptoms and mental health? • What are the best treatments to prevent or reduce negative effects of menstrual disorders or problematic symptoms on mental health? • How do findings vary by socioeconomic, demographic, global, and cultural context?
Menstrual health management	• How does the availability, accessibility, affordability, and choice of products and services to support menstrual health affect the relationship with mental health? • How do individuals with a mental health disorder manage menstruation and menstrual cycle-related experiences? • How do findings vary by socioeconomic, demographic, global, and cultural context?

## Management, administration and internal communications

4M is directed by Dr Gemma Sharp with assistance from Luana De Giorgio and support from consortium members. Relevant files are shared with members via a Google Drive. These include a members' directory (with names, contact information, affiliations, job titles, and research interests of all members), minutes from meetings, and guides for finding 4M collaborators, describing 4M in funding applications, and reporting outcomes from 4M facilitated research. Members are updated on activities and opportunities via a regular online meeting and an email newsletter. The meeting agendas are also emailed to all members and archived alongside minutes on the Google Drive.

Potential new members can express an interest in joining 4M via a Microsoft Form, which is linked from the 4M website and in a pinned Twitter post. In line with our inclusive values, all researchers, regardless of discipline, career stage, or affiliation, are accepted as members if their research aligns with the 4M vision and mission.

## Examples of activities

### Workshops and seminars

4M has run free in-person, hybrid, and online workshops for members and stakeholders, including the ideas generating and refining workshops described above, the stakeholder workshops described below, and a two-day grant writing retreat. All events have been co-led by ECRs, giving them a valuable opportunity to build their experience of designing and co-ordinating events, increase their visibility, and contribute to their CVs. All members, including ECRs, have been invited to present their work at these workshops. 4M also runs a successful online seminar series, which was established to help share research among members and more widely. We hoped that the seminar series would enhance the visibility and reach of 4M, and this seems to have been successful because our membership and Twitter following has increased since the series was established in early 2023.

### Website and social media

The 4M website (www.4Mhealth.uk) provides public-facing information about the consortium for members, other researchers, and stakeholders. It includes a blog through which relevant research is shared, for example, news from 4M members about new grants or papers, calls for research participants for relevant studies, and advertisements for our seminar series. To help share our activities more widely, and to reach and engage with new members and stakeholders, 4M also has a Twitter page (https://twitter.com/4Mhealth). All seminars in our series have been recorded and shared via our YouTube channel (https://www.youtube.com/@4Mhealth).

### Stakeholder engagement

In November 2021, 4M held a series of online workshops for stakeholders, that is, people or organisations that could be affected by or inform our research. The list of invitees was compiled by 4M members at the time. It included people who had already worked with members, as well as completely new connections. We invited individuals and organisations via email, providing a brief description of what 4M was trying to achieve and how we thought their perspective and insight would be valuable. The workshops were attended by a diverse group of stakeholders, including local school teachers, a Member of UK Parliament in the All Party Parliamentary Group (APPG) on period equality, a FemTech start-up, international menstrual activists (from Beyond Blood in India, and the Action Girls Foundation in Tanzania), charities (from the International Association for Premenstrual Disorders IAPMD, Off the Record, and Mind), policy makers (from the Department for Education and the UK Health Security Agency), academics from the Menstruation Research Network (a highly successful network of menstruation researchers, mainly from the arts and humanities, https://menstruationresearchnetwork.org.uk/about/) and representatives from the United Nations Population Fund (UNFPA, the UN's sexual and reproductive health agency). The workshops were highly generative and successful in helping us shape 4M's vision, mission and approach. We have kept in touch with many of the stakeholders and organisations and are planning future engagement events and co-produced research projects. We have also expanded our stakeholder network through our activity on Twitter and through invited presentations at events hosted by organisations including the Royal College of Psychiatrists, the Royal College of Obstetricians and Gynaecologists, and the South West International Development Network.

### Collaborative grant applications

Several project grant applications have been developed and submitted through 4M-facilitated collaborations. This includes five applications to the Medical Research Council (two successful, two unsuccessful, one in development) and four to smaller funders (one successful, two unsuccessful, one awaiting outcome). We have also put forward collaborative projects for the GW4 Doctoral Training Programme in order to attract PhD students that would be co-led by 4M researchers.

### Systematic and scoping reviews

4M is currently co-ordinating several scoping and systematic review projects. These are a necessary and useful first step to identify gaps in the literature and opportunities for 4M researchers to address them. We are currently developing protocols for reviews of prospective studies of PMS, experiences of menstruation in people with autism, and the impacts of the menopausal transition on mental disorders.

## Discussion

4M provides a useful platform to facilitate research at the intersection of menstrual and mental health. The utility of the consortium is already apparent from the wide-reaching impact we are generating. To date, we have developed and submitted several collaborative grant applications, co-supervised student projects, and been invited to take part in public engagement events and talks to stakeholder organisations. We are aware of four members (three ECRs) who were successfully promoted, having included their affiliation with 4M on their applications as evidence of interdisciplinary cross-institution research and stakeholder collaboration. Our seminar series and social media activity has been instrumental in growing our membership, which has increased from around 30 members at the start of 2023 to over 130 six months later.

Although 4M members are geographically distributed, this has not proven to be a significant challenge. The consortium was established during the Covid-19 pandemic, when most members were working from home regularly. The first workshops were online, as are our monthly meetings and our seminar series. This way of working has been a strength as it has allowed us to run workshops and grow our membership with minimum costs. Nevertheless, the value of our hybrid events cannot be overstated. These have provided an opportunity for less formal networking and collaboration, for example, allowing one-to-one or small group conversations that do not naturally occur online. Funding for these in-person events has allowed us to commit to our inclusive values by paying for childcare and travel expenses.

We have encountered some challenges to managing and sustaining the 4M consortium. By far the largest is ensuring the consortium is sufficiently resourced in terms of staff time and funding to support activities. Running the consortium, largely without administrative support, takes a substantial amount of time, which is difficult to protect in a busy academic workload without buy-out from external funding. Without funding, it can also be difficult to publish in open access journals, to run in-person networking events, to enable ECRs and researchers with childcare commitments to attend, and to pay non-academic stakeholders for their time. We are grateful to the GW4 for generously covering the direct costs needed to help establish the network. There are very few similar funding schemes to support research networks. Unfortunately, there are also limited funding opportunities for women's health research (1, 25). We are currently approaching other research funders directly and exploring opportunities to crowdfund specific activities. We heartily echo recent calls for more dedicated funding for research in women's health (2).

A second key challenge is in maintaining the interdisciplinary nature of the consortium. As with most research that aims to be interdisciplinary, there is a risk that researchers will retreat to their disciplinary silos or work only with those from closely linked disciplines (26). Interdisciplinarity is one of the key values of 4M and previous research has shown that it is positively associated with research impact (27). Strategies to maintain interdisciplinarity include: ensuring the seminar series showcases research from a breadth of disciplines, direct invites to researchers from underrepresented disciplines to join, and ensuring no technical discipline-specific language is used in our internal communications. Although this is integral to our vision and mission, maintaining interdisciplinarity is not as much of a challenge as funding and time because it is to some extent more within our control, and there is a strong willingness and desire amongst the 4M members to collaborate with others from different disciplines.

Finally, stakeholder engagement is an important part of the 4M approach, but it can be challenging to develop and maintain relationships with non-academic partners. Again, this is not, in our experience, due to a lack of enthusiasm on either side. It is mostly an issue of time (for 4M members, most of whom hold multiple competing academic roles) and funding (to pay stakeholders for their time, reimburse travel costs, and/or offer incentives). We are exploring what support is available from our institutions to assist stakeholder engagement, and we are establishing a Head of Stakeholder Engagement role to help develop this area of our activity. However, the issue of funding remains a challenge, as with no salary attached, the role-holder will need capacity within their current workload and approval from their institution to engage in work that is not (directly) generating revenue.

## Conclusion

4M was established to facilitate interdisciplinary research with the ultimate aim of ensuring that menstrual and menopausal experiences do not adversely affect mental health and social wellbeing. The consortium has grown significantly since it began and evidence of success is clear across the key components of 4M's approach (collaboration, stakeholder engagement, and ECR support). The main challenges are around securing resources to sustain the consortium and its activities, including funding for researcher time, in-person events, and to support stakeholder engagement. Ensuring research interdisciplinarity is also a key challenge and we are taking care to avoid researchers retreating back to their disciplinary silos. Overall, the 4M consortium represents an important tool for advancing knowledge and impact in menstrual and mental health. It contributes to wider attempts to increase the representation of menstrual and women's health concerns in research, and to develop evidence-based approaches to close the gender-health gap.

## Data Availability

The original contributions presented in the study are included in the article/Supplementary Material, further inquiries can be directed to the corresponding author.
